# Genome sequence of cluster A15 *Gordonia terrae* bacteriophage Nebulosus

**DOI:** 10.1128/MRA.00699-23

**Published:** 2023-09-26

**Authors:** Veronica Doyle, Gabriella Giftos, Strix Kugler, Andrew Melanson, Dominic Needham, Ryleigh Raber, Justin Solomon, Apple Webster, Melody Neely, Sally Molloy

**Affiliations:** 1 Molecular and Biomedical Sciences, University of Maine, Orono, Maine, USA; 2 The Honors College, University of Maine, Orono, Maine, USA; 3 School of Biology and Ecology, University of Maine, Orono, Maine, USA; Queens College Department of Biology, Queens, New York, USA

**Keywords:** bacteriophage, *Gordonia*, Actinobacteriophage

## Abstract

The temperate *Gordonia* phage Nebulosus was isolated from soil on *Gordonia terrae* and is a siphovirus. The genome is 52,175 bp in length, has 62% GC content, and encodes 96 protein-coding genes. Nebulosus encodes a partitioning system, ParABS, which is likely involved in lysogeny maintenance.

## ANNOUNCEMENT

**Fig 1:**
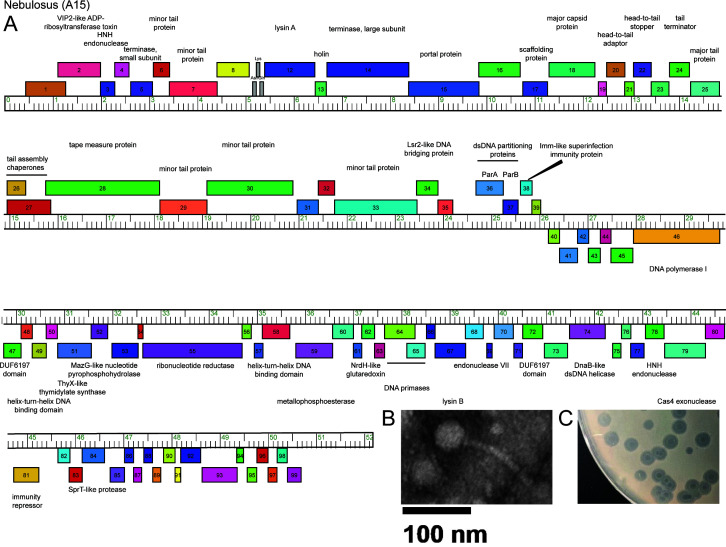
(**A**) Genome map of *Gordonia* phage Nebulosus. The ruler represents the genome coordinates in units of kilobase pairs, and the colored boxes above and below the ruler represent genes transcribed in the forward and reverse directions, respectively. Genes were assigned to a phamily using Phamerator (7) in the Actino_draft database, and different phamilies are indicated by different colors. Predicted functions were centered above and below forward- and reverse-transcribed genes, respectively. (**B**) Electron micrograph of Nebulosus. (**C**) Nebulosus plaques on a lawn of *G. terrae*.

Actinobacteriophage are diverse bacteriophages that infect Actinobacteria (1–4). Studying Actinobacteriophage increases our knowledge of phage evolution, viral defense systems, and virus-host interactions (2, 4, 5). Phage Nebulosus was isolated using direct isolation from composted soil collected on 9/1/2023 in Old Town, Maine (44.915628N, 68.69072W) (6). A soil extract was prepared in peptone-yeast extract-calcium (PYCa) medium, filtered on a 0.22-µM filter, plated with 0.5 mL of *Gordonia terrae* 3,612 on PYCa agar plates, and incubated at 30°C for 2 days. Plaques were purified after seven rounds of plaque purification using standard methods (6). On a lawn of *G. terrae*, Nebulosus forms 5-mm turbid plaques with three halo rings (Fig. 1). Nebulosus forms stable lysogens and is immune to cluster A15 phage ReMo superinfection (6). The particle morphology of Nebulosus was determined by negatively stained transmission electron microscopy of a single particle (Fig. 1). Nebulosus has a siphovirus morphology with a 120-nm-long noncontractile tail and a 46-nm-diameter icosahedral head.

A DNA phenol-chloroform extraction method was performed on a high-titer lysate (8). DNA was prepared for sequencing using the NEBNext UltraII library preparation kit (New England BioLabs, Ipswitch, MA). Sequencing on an Illumina MiSeq platform produced 163,600 single-end, 150 bp reads. *De novo* assembly and checks for completeness were performed using Newbler v2.9 and Consed v29 (9), yielding a 52,175-bp genome with a shotgun coverage of 223-fold. The genome has 62% GC content, and the genome ends with 10 bp, 3′ single-stranded overhangs (CGGGTGGTTA) (10). The genome shares >35% gene content with phages in cluster A in the Phamerator Actino_Draft database (version 521) and was assigned to subcluster A15 (4, 7).

The Nebulosus genome was auto-annotated using GLIMMER v3.02 and GeneMark v2.5 within DNA Master v.5.23.6 (http://cobamide2.bio.pitt.edu) and PECAAN (https://blog.kbrinsgd.org/) (11, 12). Translational starts were chosen based on the inclusion of GeneMark.hmm predicted coding potential and start conservation across homologs determined by BLASTp and Starterator analyses (http://phages.wustl.edu/starterator) (12, 13). Putative gene functions were predicted using BLASTp, TMHMM, and HHpred (13–15). Three tRNA genes were identified using ARAGORN v1.2.38 and tRNAscan-SE (16, 17). A map of the Nebulosus genome was prepared using Phamerator in the database Actino_Draft (version 521) (Fig. 1) (7). Nebulosus encodes 96 protein-coding genes. The genome’s left arm contains forward-transcribed assembly and structural genes (gp1–gp36) including a Vip2-like ADP-ribosyltransferase toxin (gp2), lysin A (gp12), and holin (gp13). The right arm encodes reverse-transcribed genes (gp37–gp96) with functions typical of cluster A genomes including a DNA polymerase I (gp46) and two primases (gp64 and 65). Nebulosus lacks an integrase gene but does encode ParA (gp36) and ParB (gp37) genes, which maintain stable lysogens for other cluster A phages (18).

Nebulosus and all A15 cluster members encode a gene (gp47) downstream from the DNA polymerase I (gp46) with strong HHpred hits to a domain of unknown function (DUF6197) found in a *Streptomyces kanamyceticus* kanamycin biosynthetic gene cluster (19). Nebulosus and 10 other A15 members carry a second copy of this gene (gp72). There appears to have been an INDEL in gp72 as the other 10 A15 members carry instead a longer gene with two tandem DUF6197 domains and nearly identical amino acid sequence identity to Nebulosus gp72 at the C-terminus end.

## Data Availability

Nebulosus is available at GenBank with the Accession No. OR159677 and the Sequence Read Archive (SRA) No. SRX20165775.
